# The outcomes of newly diagnosed elderly multiple myeloma patients treated at a single U.S. institution

**DOI:** 10.1002/cam4.620

**Published:** 2016-01-22

**Authors:** Lauren Bonomo, Jerry Lue, Sundar Jagannath, Ajai Chari

**Affiliations:** ^1^ Department of Medical EducationIcahn School of Medicine at Mount SinaiNew York CityNew York10029; ^2^ Division of Hematology OncologyIcahn School of Medicine at Mount SinaiGustave Levy Place, Box 1185New York CityNew York10029

**Keywords:** Chemotherapy, elderly, myeloma, neoplasms, plasma cell, United States

## Abstract

Improvements in the outcomes of elderly multiple myeloma (MM) patients have lagged behind those of transplant‐eligible patients, likely due in part to the use of less efficacious melphalan‐containing regimens. To date, there are very limited data for the outcomes of elderly MM patients in the United States (US), particularly for novel agent‐containing triplet regimens. In this retrospective study at a single U.S. institution, the outcomes of 117 consecutive newly diagnosed, symptomatic MM patients over the age of 70 were evaluated. The median age was 75 years (range 70–95) with significant baseline comorbidities including 36% cardiac and 20% renal (CrCl < 30 mL/min). The median follow‐up was 43 months and the median number of lines of therapy during the study period was 2 (1–7). Eighty‐six patients (83%) received non‐melphalan doublet, triplet, or quadruplet initial therapy, most with significant planned dose attenuations. For those treated with dose‐attenuated RVD (*n* = 34), the outcomes were particularly impressive with overall response rate (ORR), complete remission and very good partial remission (CR + VGPR), and progression‐free survival (PFS) of 94%, 65%, and 36 months, respectively, and overall survival (OS) not reached. The PFS with RVD was significantly greater than that of all other regimens (*P *= 0.030), including RD.

## Introduction

The emergence of novel treatment regimens for multiple myeloma (MM) has led to significant improvements in survival within the past decade 1. Improvements in prognosis for older patients, though still significant, have been less pronounced 2, 3. Younger MM patients do not typically receive low‐dose, oral melphalan‐based regimens because melphalan can impair the collection of autologous stem cells 4. In contrast, older patients, who are typically ineligible for autologous stem cell transplantation (ASCT) 5, almost universally receive melphalan‐based regimens in most of the world primarily because of their low cost. However, compared to the novel agents IMID and PI, melphalan is less potent, slower in onset of activity, and more myelosuppressive 6. As elderly patients diagnosed with MM will likely have more comorbidities (including impairment in cardiac, pulmonary, renal, or hepatic function) or be frailer than younger patients, these properties of melphalan could have a significant effect on both efficacy and toxicity 7. Indeed, data from the FIRST (Frontline Investigation of Lenalidomide + dexamethasone vs. Standard Thalidomide) phase III study comparing lenalidomide and dexamethasone to melphalan, prednisone, and thalidomide have already demonstrated significant improvements in progression‐free survival (PFS) and overall survival (OS) rates at 4 years 8.

To date, most studies of the efficacy of novel induction therapies have focused on younger, ASCT‐eligible patients 9, 10. Those that have focused on the elderly, such as the FIRST study, have been conducted outside of the United States, where conventional melphalan‐based induction therapies remain predominant for ASCT‐ineligible patients. There is currently a scarcity of data on how novel triplet induction regimens fare in comparison to conventional induction therapies for this group of patients. Furthermore, there are no data comparing the outcomes of doublet and triplet novel agent induction therapies for the elderly. In this retrospective study, we examined the outcomes of consecutive newly diagnosed elderly MM patients treated at a single institution in the United States, including many who received novel doublet or triplet initial therapy.

## Methods

We retrospectively reviewed the records of 133 consecutive patients over the age of 70 at the time of initial diagnosis with symptomatic MM with at least 1 year of follow‐up. The patients were seen at St. Vincent's Catholic Medical Center and upon closure at the Mount Sinai Hospital, between 1998 and 2013. Patients were treated based on practicing physician preference and were excluded if their medical records contained insufficient treatment information. Of the 133 consecutive charts reviewed, 16 were eliminated due to incomplete records, leaving 117 eligible patients.

High‐risk MM by cytogenetics was defined as deletion 13. The following were considered high risk by FISH: deletion of 17p, deletion of 1p, amplification of 1q, t(14:16), and t(14:20). Intermediate risk by cytogenetics was hypodiploidy, with t(4:14) by FISH 11. Response to treatment was assessed according to the International Myeloma Working Group (IMWG) uniform response criteria 12. This study was approved by the Mount Sinai School of Medicine's institutional review board.

PFS and OS are calculated from the first day of induction therapy using the Kaplan–Meier method. Comparisons at the univariate level were made using the log‐rank test, and multivariate analysis was performed using a Cox proportional hazards model. Statistical analyses were performed using IBM SPSS Statistics, version 20, Armonk, NY, United States.

## Results

**Figure 1:**
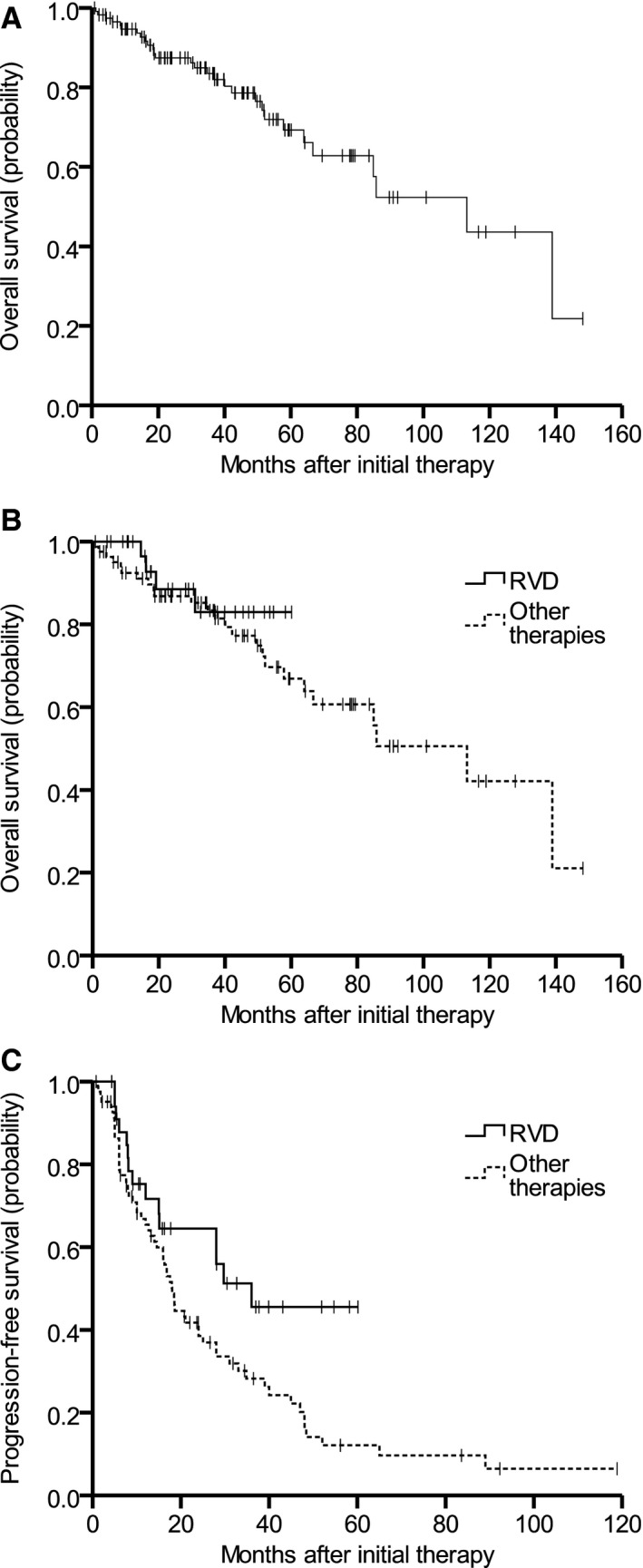
(A) Kaplan–Meier analysis of overall survival (OS) for all subjects. (B) OS for RVD versus all other regimens. (C) Progression‐free survival (PFS) for RVD versus all other regimens.

Of the 117 patients who met the eligibility criteria, the median age was 75 years (range 70–95). There were significant baseline comorbidities including 36% cardiac, 20% renal (CrCl < 30 mL/min), and 5% pulmonary disease. Of the 79 patients with ISS information, 36% were stage III, and of the 68 patients with cytogenetics/FISH information, 9% were high risk. The median follow‐up was 43 months (1–154 months), and the median number of lines of therapy was 2 (1–7). Impressively, the median OS for the entire group patients was 113 months (Fig. 1).

Excluding four patients of the 117 who received corticosteroids alone, 95 patients out of 113 (84%) received nonmelphalan doublet, triplet, or quadruplet initial therapy (Table 1). For those treated with RD plus bortezomib (RVD), despite significant dose attenuations (78% received 1.3 mg/m^2^ bortezomib SQ once weekly and 76% received lenalidomide 15 mg daily for 21 days), the outcomes were particularly impressive with overall response rate (ORR), CR + VGPR, and PFS of 94%, 65%, and 36 months, respectively, and OS not reached (Table 2). Only three patients (2.5%) had documented NCI CTC Grade 3 or 4 toxicities. Conventional MM prognostic variables were balanced in triplet versus doublet groups; however, the prevalence of high‐risk cytogenetics/FISH was too low to make meaningful comparisons. The PFS with RVD was significantly greater than that of all other regimens (*P *= 0.030, Fig. 1), including RD (*P *= 0.047).

**Table 1:** Patient characteristics

	*n* (total = 117)
Median age, years (range)	75 (70–95)
Gender (% male)	57
Comorbidities (%)	
Renal disease	
CrCl < 30 mL/min	23 (19)
CrCl between 30 and 60 mL/min	55 (44)
Cardiac disease	43 (36)
CAD	12 (10)
CHF	25 (21)
Arrhythmia	22 (19)
Pulmonary disease	6 (5)
Myeloma isotype (%)	
IgG	56 (48)
IgA	25 (21)
IgM	1 (1)
Light chain	28 (24)
Unknown	7 (6)
Durie‐Salmon stage (%)	
I	16 (14)
II	37 (32)
III	40 (34)
Unknown	24 (21)
ISS stage (%)	
I	32 (27)
II	24 (21)
III	32 (27)
Unknown	29 (25)
Cytogenetics/FISH (%)	
High risk	6 (5)
Intermediate risk	13 (11)
Standard risk	49 (42)
Unknown	49 (42)

**Table 2:** Response rates, PFS, and OS for all groups with 10 or more patients

Therapy	*N*	ORR (%)	VGPR + CR^a^ (%)	Median PFS (months)	Median OS (months)
Doublet	42	71	48	18.5	84.9
VD	17	65	47	24	NR
RD	15	67	40	18.5	66.7
TD	4	–	–	–	–
MP	3	–	–	–	–
VC	2	–	–	–	–
CP	1	–	–	–	–
Triplet/quadruplet	71	85	51	21	NR
RVD	34	94	65	36	NR
With maintenance	21	–	–	NR	NR
Without maintenance	13	–	–	9	NR
VCD	13	69	31	16.8	NR
VMP	11	81	45	14	NR
VCDT	5	–	–	–	–
MPT	4	–	–	–	–
VTD	2	–	–	–	–
CTD	2	–	–	–	–
All patients^b^	117	78	48	18.6	113

ORR, overall response rate; VGPR + CR, very good partial response + complete remission; PFS, progression‐free survival; OS, overall survival; VD, bortezomib, dexamethasone; RD, lenalidomide, dexamethasone; TD, thalidomide, dexamethasone; MP, melphalan, predisone; VC, bortezomib, cyclophosphamide; CP, cyclophosphamide, prednisone; RVD, lenalidomide, bortezomib, dexamethasone; +/− maintenance with/without maintenance; VCD, bortezomib, cyclophosphamide, dexamethasone; VMP, bortezomib, melphalan, prednisone; VCDT, bortezomib, cyclophosphamide, dexamethasone, thalidomide; MPT, melphalan, prednisone, thalidomide; VTD, bortezomib, thalidomide, dexamethasone; CTD, cyclophosphamide, thalidomide, dexamethasone.

a CR unconfirmed.

b Includes four patients whose regimens were corticosteroid‐only.

The median number of cycles of RVD administered prior to beginning maintenance was 5 (range 3–9). After completion of these cycles, bortezomib was typically omitted and RD was weaned down to a minimal dose (R to 10 mg and D to off) as a maintenance strategy. The CR + VGPR rates upon completion of induction in RVD (*n *= 13) only versus RVD with maintenance (*n *= 21) were 62% and 67%, respectively. Of note,12 out of 34 (34%) patients treated with VRD had unconfirmed CRs (i.e. confirmatory bone marrow biopsies were not performed) and 10 of 34 (29%) had VGPRs. Impressively, the median PFS of the 21 patients receiving lenalidomide maintenance was not reached despite a median follow‐up of 37.0 months versus a median PFS of 29.0 months for those not receiving lenalidomide maintenance.

The toxicities noted with RVD included peripheral neuropathy, rash, anemia, and thrombocytopenia. Two (5.9%) of these patients had documented grade 2 neuropathy with pain. While 29% of patients had dose reductions, only 8.8% (*n *= 3) required unplanned dose attenuations due to toxicity; two of these were a result of neuropathy with pain and one was due to an exacerbation of Parkinson's symptoms. The remainder of dose attenuations were made after achieving disease control. Moreover, no patients initially treated with RVD discontinued therapy entirely as a result of toxicity.

The most common (greater than 5 patients) salvage regimens were RVD (21.7%), RD, VCD, and clinical trials. A total of seven and five patients received carfilzomib or pomalidomide during the study period. The most common salvage regimen at first relapse was RVD.

## Discussion

This study is the largest to date that examines outcomes of elderly MM patients from the United States where the majority of subjects received non‐melphalan containing initial regimens. Because so many of our patients (86%) received non‐melphalan induction therapy, we are unable to make a direct comparison between melphalan and non‐melphalan frontline regimens. However, similar to the findings of the FIRST study, outcomes of our non‐melphalan‐based induction regimens compare favorably to published historical controls, where the average RR, CR, PFS, and OS from the five large studies on MP and MPT were 39%, 2.6%, 15 and 30 months and 66%, 11%, 20.7 and 39.2 months, respectively (Table 3) 10.

**Table 3:** Outcomes of published phase III studies in elderly patients with MM

Regimen [reference]	Maintenance?	*n*	PR (%)	CR (%)	Median PFS (months)	Median OS (months)	3‐year OS (%)
MP 4	No	196	35	2	17.8	33.2	–
MP 10	No	179	40	4	14	32	–
MP 13	No	173	45	NR	11	31	–
MP 6, 14	No	164	47.6	3.7	14.5	47.6	–
MP 15	No	116	31	1	18.5	29.1	–
MPT 16	Yes	184	57	13	15	29	–
MPT 13	Yes	171	66	NR	15	40	–
MPT 6, 14	Yes	167	76	15.6	21.8	45	–
MPT 4	No	125	76	13	27.5	51.6	–
MPT 15	No	113	62	7	24.1	44	–
MPV 17	Yes	167	73	34	NR^b^	–	NR^b^
MPV 18, 24	Yes	130	80	20	32	–	74
MPV 19, 20	No	344	71	30	24	–	68.5
MPV 21	No	257	81	24	a	–	87
VTP 18	Yes	130	81	28	25	–	65
VD 17	Yes	168	71	31	NR^b^	–	NR^b^
VTD 17	Yes	167	79	38	NR^b^	–	NR^b^
RD 8	Yes	121	–	–	25.5	NR^c^	58^d^

PR, partial remission; CR, complete remission; Med. PFS, median progression‐free survival (months); Med. OS, median overall survival (months); 3‐yr OS, three‐year overall survival (%); MP, melphalan, prednisone; VD, bortezomib, dexamethasone; RD, lenalidomide, dexamethasone; MPT, melphalan, prednisone, thalidomide; MPV, melphalan, prednisone, bortezomib; VTP, bortezomib, thalidomide, prednisone; VTD, bortezomib, thalidomide, dexamethasone.

a Three‐year PFS = 41%.

b At 49 weeks.

c At 37 weeks.

d Four‐year OS.

The outcomes noted with RVD, the most frequently used triplet regimen in our cohort, merit special attention. This regimen is increasingly used in younger patients based on published results 22. While elderly patients with symptomatic MM deserve the same rapid time to response, depth of response, PFS, and OS benefits associated with this regimen that younger patients have obtained, some feel that a triplet regimen would be more toxic in elderly patients. Here, not only do we show that dose‐attenuated triplet regimens were associated with similar outcomes seen with younger patients treated with full dose RVD, there were very few additional unplanned dose reductions for toxicity with this regimen. Indeed we have shown similar efficacy results in an equally (if not more) frail AL amyloid population 23. The use of triplet regimens in the elderly is further supported by recent data showing improved PFS and OS in patients first treated with VMP 24.

Furthermore, once patients achieved the desired depth of response (CR or VGPR), it was possible to dose attenuate the triplet regimen. The parenteral agent was typically omitted first with subsequent maintenance on the oral lenalidomide and dexamethasone, with progressive dose attenuations on these agents as well until only a low dose of maintenance lenalidomide is attained. Importantly, this approach allows for retreatment with RVD at the time of relapse. Of the 21 patients treated with RVD who went on to maintenance lenalidomide, the median PFS was not reached despite a median follow‐up of 37.0 months. Once the prospective phase 1/2 study of RVD Lite is completed by those at the Dana‐Farber Cancer Institute (NCT01782963) which entails lenalidomide days 1–21, bortezomib weekly (intravenously for cycle 1 and thereafter subcutaneous), and dexamethasone either weekly or twice weekly depending on age, confirms our findings, a phase III study comparing RVD Lite to RD could then definitely resolve the risk/benefit ratio of RVD versus RD in the elderly population.

An equally striking finding was that the median OS for all elderly U.S. patients in this study was 113 months, with a 95% confidence interval of 70.0–156.3 months. Even the lowest end of this confidence interval shows a marked improvement in our U.S. patients compared to previously published data from Europe and Asia. Given the outcomes seen with RVD in our study, the increased efficacy of newer induction regimens could result in improvements in OS for elderly patients comparable to those already being seen in the younger population.

However, it is important to note that elderly patients may not need the same dosages of the novel agents as younger patients. The prescribing information for lenalidomide indicates dose modifications for CrCl < 50, which likely applies to at least 30% of those over the age of 70 25. Indeed, 85% of our cohort was receiving planned dose‐attenuated therapy. Equally important, the improved OS outcomes in this study are likely also due in part to the readily available salvage regimens in the United States.

There are several limitations of this study. One of the main factors predictive of outcome in elderly MM patients is frailty, and several indices have been studied to measure it. Unfortunately, these indices must be performed at the time of diagnosis, and were not available in this retrospective study. It is important to note that frailty has been shown to be a determinant of prognosis independent of treatment regimen 26. Additional data that were difficult to collect in this retrospective study were the cytogenetics/FISH status in these elderly patients and confirmation of a CR. The former is due to initial bone marrows often being performed outside our center and not being repeated for those patients not enrolled in clinical trials. The absence of a bone marrow to confirm a CR is common outside of a clinical trial. These limitations could be addressed in prospective clinical trials.

Selection bias must be considered in a retrospective, single institution study such as this one. It is not possible to control which patients are prescribed specific regimens, although we did not observe any particular patterns in the choice of treatments, as all other prognostic variables (e.g., comorbidities, ISS stage, FISH) seemed comparable across groups, and no pattern was observed in the ratio of triplet to doublet therapy over time. Finally, the inherent limitations of a retrospective study preclude definitive causal attribution, particularly given a possible referral bias for patients seen at an academic referral center. However, the inclusion of all consecutive patients (including many with comorbidities that would not meet standard clinical trial eligibility criteria) provides important real world data.

There have been numerous developments in the field of geriatrics, including the burgeoning subspecialty of geriatric oncology. The application of the principles of frailty indices, pharmacokinetic‐guided planned dose modifications, and prospective studies in the elderly leading to global drug availability will be essential to the ensuring that the benefits of novel agents reach all patients, regardless of age and geographic location.

## Conflict of Interest

SJ has served on advisory boards for Bristol‐Myers Squibb; Celgene Corporation; and Sanofi‐aventis. AC has served on advisory boards for and received research funding from Celgene, Millenium‐Takeda, Onyx, and Array BioPharma. He has given educational lectures for Celgene.
